# Hydrogen Sulfide Inhibits Ferroptosis in Cardiomyocytes to Protect Cardiac Function in Aging Rats

**DOI:** 10.3389/fmolb.2022.947778

**Published:** 2022-07-22

**Authors:** Zihui Liang, Yuxin Miao, Xu Teng, Lin Xiao, Qi Guo, Hongmei Xue, Danyang Tian, Sheng Jin, Yuming Wu

**Affiliations:** ^1^ Department of Physiology, Hebei Medical University, Shijiazhuang, China; ^2^ Hebei Collaborative Innovation Center for Cardio-Cerebrovascular Disease, Shijiazhuang, China

**Keywords:** aging, ferroptosis, cardiac dysfunction, hydrogen sulfide, iron metabolism

## Abstract

Aging contributes significantly to cardiovascular diseases and cardiac dysfunction. To explore the reasons for the decline in cardiac function in the elderly, we collected clinical data and blood samples from 231 individuals. Our results indicated that aging was accompanied by a decline in cardiac function and remodeling of the left ventricle, and cardiac function was negatively correlated with age. Serum hydrogen sulfide (H_2_S) decreased, while serum malondialdehyde (MDA) and iron increased with aging in healthy individuals. A rat model of aging and iron overload was constructed for *in vivo* research. In the animal model, we found that the expression of endogenous H_2_S-producing enzymes decreased, and endogenous H_2_S levels decreased, while oxidative stress levels rose. The regulation of iron metabolism and the maintenance of iron homeostasis declined. The accumulation of MDA and iron led to ferroptotic cell death and subsequent myocardial injury and deterioration. A high-iron diet accelerated the aging process and death in rats. The decline of cardiac function in aging rats and iron-overload rats may be caused by cardiomyocyte ferroptosis. Exogenous H_2_S enhanced the expression of endogenous H_2_S synthase, promoted endogenous H_2_S production, regulated iron metabolism, and reduced oxidative stress levels. The protective effects of H_2_S on cardiac function in aging rats and iron-overload rats may be partly due to the inhibition of cardiomyocyte ferroptosis. We demonstrated that cardiac dysfunction associated with aging was closely related to decreased endogenous H_2_S levels and cardiomyocyte ferroptosis. H_2_S-regulated iron metabolism reduced oxidative stress levels in cardiomyocytes, inhibited cardiomyocyte ferroptosis, and protected cardiac function in aging rats.

## Introduction

Aging is a dominant risk factor for cardiovascular diseases, including hypertension, cardiac hypertrophy, and heart failure, which ultimately lead to increased mortality (Timmers et al., 2020). Aging hearts exhibit unique histological and physical features**,** including increased cell death, increased cardiomyocyte volume, and collagen fiber accumulation (Julian et al., 2019). Given the dramatically aging population, age-related cardiac dysfunction represents one of the greatest challenges confronting global healthcare today.

The main reason for cardiac dysfunction due to aging is cardiomyocyte death. At least 12 forms of regulated cell death have been described, of which seven have been implicated in the cardiovascular system, including apoptosis, mitochondrial-permeability-transition-driven necrosis, necroptosis, pyroptosis, parthanatos, autophagy-mediated cell death, and ferroptosis (Galluzzi et al., 2015; Galluzzi et al., 2018). Ferroptosis is an iron-dependent form of regulated cell death characterized by the accumulation of lipid peroxide to lethal levels, resulting in oxidative damage to cell membranes. Ferroptosis differs from apoptosis, necroptosis, and autophagy in several aspects (Dixon et al., 2012; Stockwell et al., 2017)**.** Ferroptosis plays critical roles in cardiomyopathy, myocardial infarction, ischemia/reperfusion injury, and heart failure (Park et al., 2019; Tang et al., 2021; Tian et al., 2021; Zheng et al., 2021; Lin et al., 2022).

Ferroptosis triggered by excess iron causes cardiomyocyte death by regulating iron metabolism. Understanding the signaling pathways preventing iron-mediated cell death will provide new approaches for treating patients with acute myocardial infarctions (Baba et al., 2018). Therefore, the ability of H_2_S to regulate iron metabolism and homeostasis deserves our attention (Zhang et al., 2019). H_2_S is an important gaseous signaling molecule. H_2_S exerts protective effects, including vasoactive effects and anti-inflammatory and antioxidant properties (Perna et al., 2010). H_2_S contributes to ischemic postconditioning-induced cardioprotection in aging hearts and cardiomyocytes (Li et al., 2016a). Our research group demonstrated that exogenous H_2_S restored the diurnal variations in cardiac function in aging mice (Zhang et al., 2021). Decreased antioxidative stress capabilities can cause ferroptosis in cardiomyocytes, and decreasing iron accumulation and/or inhibiting lipid peroxidation is cardioprotective during both acute and chronic cardiac ischemia/reperfusion-induced cardiomyopathy (Fang et al., 2019). Thus, regulating the expression of antioxidative stress-related proteins, such as NRF2 and GPX4, can inhibit cardiomyocyte ferroptosis (Bai et al., 2018). These findings indicate that ferroptosis is a potential target for protection against cardiomyopathy. Therefore, the impact of H_2_S on iron accumulation and the antioxidative stress characteristics contributing to decreased cardiomyocyte ferroptosis are worth exploring.

The specific form of cell death in cardiomyocytes associated with aging in humans remains unclear. Furthermore, the different effects of aging on human cardiac function during health and disease have not been elucidated. The world’s population is aging, highlighting the need to understand the impact of aging on cardiac function. However, the occurrence of cardiomyocyte ferroptosis in healthy individuals and the effects of endogenous H_2_S levels on cardiac aging and ferroptosis in cardiomyocytes are not clear. With this in mind, the aim of the present research was to investigate whether cardiac dysfunction caused by aging is related to cardiomyocyte ferroptosis and changes in endogenous H_2_S levels. Potential mechanisms will be explored.

## Materials and Methods

### Clinical Sample Data and Blood Sample Collection

Clinical data and blood samples were collected from 231 individuals undergoing medical examination at The Third Hospital of Shijiazhuang in China from July 2021 to October 2021. Participants were aged 20–75 years and included 135 males and 96 females. Participants were divided into the following age groups: 20–29 years, 30–39 years, 40–49 years, 50–59 years, and 60 + years. The inclusion criteria were as follows: 1) denial of a medical history of coronary heart disease, cerebral infarction, cerebral hemorrhage, hypertension, or diabetes; 2) denial of a medical history of hepatitis or tuberculosis; and 3) denial of a medical history of surgery and blood transfusion. The general condition and medical history of all participants were recorded. Blood samples were prospectively obtained from participants at admission. After centrifugation for 10 min at 3,500 rpm, the supernatants were frozen at −80°C. All participants provided written informed consent prior to the study. The protocol was approved by the Ethics Committee of the Hebei Medical University (Shijiazhuang, China) and performed in accordance with all applicable laws, regulations, and guidelines in China (including good clinical practice guidelines), which are related to the protection of human subjects as volunteers. The study was conducted according to the published regulations of the Declaration of Helsinki.

### Animals and Experimental Protocol

The animal study was reviewed and approved by the Animal Management Rule of the Ministry of Health, People’s Republic of China (documentation number 5, 2001) and the Animal Care Committee of Hebei Medical University. The protocols and procedures performed were in compliance with the Guide for the Care and Use of Laboratory Animals (NIH Publication No. 85–23, revised 2011). Animals were obtained from the Animal Center of Hebei Medical University (Shijiazhuang, China) and housed in plastic cages in a room with a controlled humidity of 50–65%, at a temperature of 20–25°C and on a regular 12 h light and dark cycle (lights on from 8:00 to 20:00). They were fed on standard rat chow and tap water *ad libitum*.

### Rat Model of Aging

Twenty-four Sprague–Dawley (SD) rats were divided into three groups (*n* = 8/group): an old group, an old + NaHS group, and a young group. The old groups were composed of 15-month-old male SD rats randomly divided into treated and untreated groups (+/−NaHS), and the young group was composed of 8-week-old male SD rats. The old + NaHS group received NaHS (100 μmol/kg, i.p./d) for 3 months. The young and untreated old groups received an equal volume of saline (i.p./d) for 3 months.

### High-Iron Diet Aging Rat Model

Thirty-six 15-month-old male SD rats were randomly divided into three groups (*n* = 12/group): an old + normal-iron diet (NID) group, an old + HID group, and an old + HID + NaHS group. The old + NID group received an NID for 3 months, and the old + HID groups received an HID for 3 months. The old + HID + NaHS group received the HID and NaHS (100 μmol/kg, i. p./d) for 3 months.

### HID Young Rat Model

Twenty-four 8-week-old male SD rats were randomly divided into three groups (*n* = 8/group): an NID group, an HID group, and an HID + NaHS group. The NID group received NID for 3 months, and the HID group received HID for 3 months. The HID + NaHS group received HID and NaHS (100 μmol/kg, i. p./d) for 3 months.

At the end of the study protocol, cardiac function was determined by echocardiography, and hemodynamic parameters were measured using the Power Lab (ML4818) Data Acquisition System (ADInstruments, Australia). Blood samples were collected, immediately centrifuged for 10 min at 3,500 rpm, and frozen at−80°C. The heart was rapidly removed and frozen at −80°C.

### HID Preparation

A chow diet containing 0.241% elemental iron (Research Diets, D08080405, Beijing KeAoXieLi Feed Co., Ltd., China) was prepared. In brief, 1 kg of a chow diet was ground and combined with 2.41 g of carbonyl iron. Then, 1.2 L of deionized water was added to the HID. After mixing, the iron loading diet was molded and baked at 80°C for 24 h in a hot air oven.

### Echocardiographic Assessment

The participants took the supine position, and after an appropriate amount of couplant was applied to the precordial area, the participants were examined with an M-mode ultrasound. Cardiac functional parameters were evaluated, including left ventricular ejection fraction (LVEF), left ventricular fractional shortening (LVFS), stroke volume (SV), left ventricular internal dimension (LVID), interventricular septum (IVS), left ventricular anterior wall (LVAW), left ventricular posterior wall (LVPW), left atrial volume, maximum flow velocity of the mitral valve in early diastole (E′ wave), maximum flow velocity of the mitral valve in late diastole (A′ wave), mean diastolic velocity of mitral annulus in diastole (e'), and heart beats per minute (BPM). The cardiac output (CO), cardiac index (CI), E′ wave/A′ wave (E/A), E/e', and left atrial volume index (LAVI) were calculated from the measured cardiac parameters from five consecutive cardiac cycles and averaged.

Rats were anesthetized with 2–3% inhaled isoflurane (RWD Life Science Co., Ltd., Shenzhen) and immobilized on the operating table. To evaluate the cardiac function, mouse two-dimensional echocardiography was performed using a Vevo 2,100 ultrasound device (FUJIFILM Visual Sonics Inc., Toronto, Canada). Parasternal long axis and short axis images were obtained in two-dimensional and M-modes for quantification. Left ventricular anterior wall diastole (LVAW’ d) and left ventricular posterior wall diastole (LVPW’ d) were measured on the parasternal left ventricular long axis view. Using the PW-Doppler mode, E′ wave and A′ wave were measured. LVEF, LVFS, and CO were calculated using computer algorithms. Measurements were averaged over five consecutive cardiac cycles in a blinded manner.

### Hemodynamics Evaluation

Rats were anesthetized with pentobarbital sodium (30 mg/kg, i. p.), and the carotid artery was catheterized. A pressure transducer (ML4818) connected to the Power Lab apparatus (AD Instruments, Australia) was used to measure blood pressure and cardiac function. A polyethylene catheter was introduced from the right carotid artery, and a catheter (retrograde) was introduced through the aortic valve to reach the left ventricular (LV) cavity. LV pressure was recorded for 15–30 min. All data were recorded and analyzed by Lab Chart 7 software. The maximum rates of the left ventricular pressure rise and fall (+dp/dt_max_ and −dp/dt_max_) and left ventricular end diastolic pressure (LVEDP) were acquired from the pressure waves. The rats were euthanized after determining pressure, and blood and heart tissues were collected and stored.

### Blood Analysis

Serum creatinine (CRE), blood urea nitrogen (BUN), alanine aminotransferase (ALT), and aspartate aminotransferase (AST) levels were measured in participants. Plasma lactate dehydrogenase (LDH), creatine kinase (CK), and CK-myocardial band (CK-MB) levels were measured in rats. The blood parameters were determined using an automatic biochemical analyzer (AU5800, Beckman, United States).

### Measurement of H_2_S Levels

Serum and plasma H_2_S concentrations were detected by liquid chromatography-tandem mass spectrometry. The blood samples were centrifuged at 3,500 rpm for 15 min, and the serum was tested. The frozen heart tissue samples were thawed on ice and weighed. Saline was added to the heart tissue at a ratio of weight (g): volume (ml) = 1:9, and the tissue was mechanically homogenized on ice. The homogenized tissue was centrifuged at 2,500 rpm for 10 min, and the supernatant was collected. Na_2_S at different concentrations (0.00, 0.08, 0.16, 0.31, 0.63, 1.25, 2.50, 5.00, 10.00, 20.00, and 40.00 μmol) were used to generate a standard curve. Samples (60 μL) were added to the EP pipe filled with nitrogen, and 140 μL of tris HCl buffer solution (pH = 10.1) and 60 μL of MBB derivatization reagent were added. After 120 min of light-shielded reaction at 50°C, 100 μL of SSA solution was added to terminate. The precipitated protein was centrifuged at 12,000 rpm at 4°C for 10 min, and the supernatant was collected and filtered through a 0.22-μm filter membrane. The filtered solution was injected into the sample injection bottle and put it into the automatic sample injector for sample injection detection. The Na_2_S standard curve was used to calculate the H_2_S concentration in serum (unit: μmol/L), plasma (unit: μmol/L), and cardiac tissues (unit: μmol/g protein).

### Measurement of MDA, Iron, and GSH Levels

The levels of MDA in the serum, plasma, and cardiac tissues were measured using a malondialdehyde (MDA) Assay Kit (A003-2-2, JianCheng, Nanjing, China). Frozen serum, plasma, and heart samples were thawed on ice. The heart tissue was weighed, and normal saline was added at a ratio of weight (g): volume (ml) = 1: 9. After mechanical homogenization on ice, the sample was centrifuged at 500 rpm for 10 min. The protein concentration in the supernatant was determined using the BCA assay. The reagents were prepared according to the MDA kit instructions and kept on ice away from light. The sample (0.2 ml), reagent 1 (0.2 ml), reagent 2 (3 ml), and reagent 3 (1 ml) were combined in the sample test samples, and standard (0.2 ml), reagent 1 (0.2 ml), reagent 2 (3 ml), and reagent 3 (1 ml) were added to the standard test tubes. Absolute ethanol (0.2 ml), reagent 1 (0.2 ml), reagent 2 (3 ml), and reagent 3 (1 ml) were combined in the blank tube, and sample (0.2 ml), reagent 1 (0.2 ml), reagent 2 (3 ml), and 50% glacial acetic acid (1 ml) were added to the control tube. After mixing, the samples and controls were boiled for 40 min and then cooled. After centrifuging at 3,500 rpm for 20 min, the absorbance of the supernatant (1 ml) was measured at 532 nm. MDA levels of each sample were calculated according to the formula from the manufacturer.

The levels of iron in the serum, plasma, and cardiac tissues were measured using Iron Assay Kits (A039-1-1 and A039-2-1, respectively, Jiancheng, Nanjing, China). The frozen samples were thawed on ice. The heart tissue was weighed, and normal saline was added at a ratio of weight (g): volume (ml) = 1:9. After mechanical homogenization on ice, the samples were centrifuged at 2,500 rpm for 10 min. The protein concentration in the supernatant was determined using the BCA assay. The reagents were prepared according to the instructions of the iron determination kits. Samples were boiled for 5 min, cooled, and centrifuged at 3,500 rpm for 20 min. The absorbance of supernatants (1 ml) was measured at 520 nm. Iron levels were calculated according to the formula from the kit.

The levels of GSH in cardiac tissues were measured using a GSH Assay Kit (A006-1-1, Jiancheng, Nanjing, China) according to the manufacturer’s instructions. The frozen heart tissue was thawed on ice. The heart tissue was weighed, and normal saline was added at a ratio of weight (g): volume (ml) = 1: 9. After mechanical homogenization on ice, the samples were centrifuged at 2,500 rpm for 10 min. The protein concentration in the supernatant was determined using the BCA assay. The reagents were prepared according to the instructions of the GSA Assay Kit. The absorbance of each sample was measured at 420 nm, and GSH levels were calculated according to the formula in the instructions of the kit.

### Measurement of the Reactive Oxygen Species Level

ROS levels in cardiac tissues were measured by staining the fresh frozen sections with DHE. Fresh cardiac tissues were mounted using Tissue-Tek O.C.T. Blocks were sectioned into 5-μm thick slices, washed twice with phosphate-buffered saline, and incubated for 30 min with DHE (10 μmol/L). The resulting color reaction was immediately measured with a fluorescence microscope (Leica, Germany).

### Histopathological Analysis

Cardiac tissues were collected from five mice in each group. The tissue was fixed in 10% neutral buffered formalin, embedded in paraffin, and then sectioned at 4-µm thickness. Cardiac sections were deparaffinized at 60°C for 1 h and hydrated in distilled water. After hematoxylin and eosin (HE) staining, the tissue sections were examined using an optical microscope (OLYMPUS) at ×200 magnification. The proportion of inflammatory cells was determined as the percentage of inflammatory cells to the total cardiomyocytes. To evaluate myocardial fibrosis, the tissue sections were stained using a Masson’s trichrome staining kit (BASO, BA-4079). Tissue sections were examined using an optical microscope (OLYMPUS) at ×100 magnification. The myocardium was stained red, the nucleus was stained black, and collagen fibers were stained blue. The collagen volume fraction was determined as the percentage of collagen (blue-stained area) to the total cardiac tissue area. Using Prussian blue iron staining, iron accumulation in cardiac tissues was evaluated, and equal volumes of potassium ferrocyanide solution and hydrochloric acid solution were mixed to make a working iron stain solution. The cardiac tissues were then incubated with the working solution for 3 min. The tissue sections were examined using an optical microscope (OLYMPUS) at ×400 magnification. The cardiac iron density was determined as the percentage of blue-stained cells to the total cardiomyocytes. All data were processed and analyzed by ImageJ software (1.52, NIH, United States).

### Electron Microscopy

To detect changes in the mitochondrial morphology of cardiomyocytes during aging, electron microscopy was conducted. Cardiac tissues were fixed at 4°C with 2% glutaraldehyde in a 0.1 M sodium cacodylate buffer and post-fixed for 1 h on ice with 1% osmium tetroxide. The slices were stained with uranyl acetate and observed under an electron microscope.

### Western Blotting

Frozen heart tissues were lysed mechanically in cold RIPA buffer. Proteins were extracted and quantified using a BCA protein assay kit (Best Bio, Shanghai, China). The proteins were electrophoretically separated using SDS-PAGE and transferred to a polyvinylidene difluoride membranes (Millipore, United States). The membranes were blocked with 3% BSA for 1.5 h at room temperature, and antigens were detected using the following antibodies at 4°C overnight, CSE (1:1,000, Proteintech, United States), CBS (1:1,000, Proteintech, United States), 3-MST (1:5,000,SantaCruz, United States), P53 (1:2000, Proteintech, United States), P21 (1:2000, Proteintech, United States), NRF2 (1:2000, Proteintech, United States), SLC7A11 (1:2000, Proteintech, United States), ACSL4 (1:2000, Abcam, United States), GPX4 (1:2000, Proteintech, United States), FPN1 (1:1,000, Proteintech, United States), FTH (1:1,000, Immunoway, United States), TRF1 (1:1,000, Abcam, United States), and GAPDH (1:5,000, Proteintech, United States). The blots were incubated with a horseradish peroxidase-conjugated anti-rabbit (Proteintech, United States) or anti-mouse (Proteintech, United States) secondary antibody at room temperature for 1.5 h. All antibodies were diluted with TBST. Blot bands were visualized by enhanced chemiluminescence and detected by ImageJ software (1.52, NIH, United States).

### Statistical Analyses

Statistical analyses were performed by SPSS 21 software (SPSS Inc., Chicago, IL, United States). Data are presented as the mean ± standard error of the mean, and independent t-tests were used to compare two groups. The results of more than three groups were compared using a one-way analysis of variance followed by the LSD test. Spearman correlation analysis was performed. *p* < 0.05 was considered statistically significant.

## Results

### Cardiac Function Negatively Correlates With Age in Healthy Participants

**FIGURE 1:**
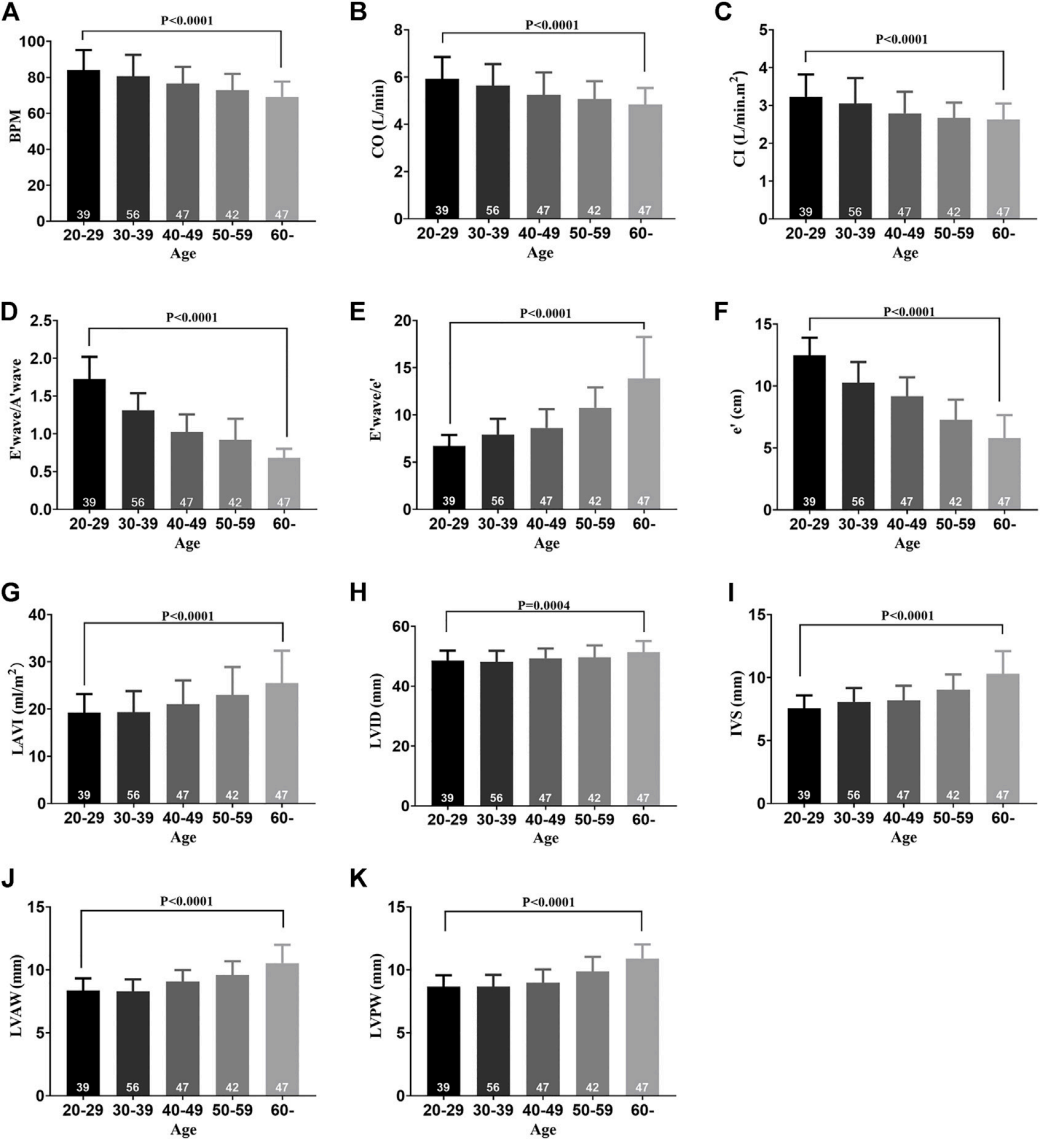
Cardiac function decline and left ventricle remodeling with aging in healthy participants. **(A)** BPM of five groups. **(B)** CO of five groups. **(C)** CI of five groups. **(D)** E′ wave/A′ wave ratios of five groups. **(E)** E′ wave/e' ratios of five groups. **(F)** e' of five groups. **(G)** LAVI of five groups. **(H)** LVID of five groups. **(I)** IVS of five groups. **(J)** LVAW of five groups. **(K)** LVPW of five groups. Results are means ± SEM. A *p*-value of <0.05 was considered significant. BPM: beat per minute. CO: cardiac output. CI: cardiac index. E’ wave: maximum flow velocity of mitral valve in early diastole. A’ wave: maximum flow velocity of mitral valve in late diastole. e’: mean diastolic velocity of mitral annulus in diastole. LAVI: left atrial volume index. LVID: left ventricular internal dimension. IVS: interventricular septum. LVAW: left ventricular anterior wall. LVPW: left ventricular posterior wall.

The characteristics of the 231 healthy participants are shown in Table 1. No significant differences in general characteristics were detected between the five groups. According to the echocardiographic measurements, the values of EF%, FS%, and SV did not decrease with aging (Supplementary Figures S1A–C), but the BPM decreased with the progress of aging, indicating that the BPM negatively correlated with age (Figure 1A). The CO and CI decreased with the progress of aging, indicating that the cardiac systolic function negatively correlated with age (Figures 1B,C). The values of E/A, E/e', e', and LAVI were statistically different between the 60 + age group and the 20–29 age groups, indicating that the cardiac diastolic function negatively correlated with age (Figures 1D–G). LVID of healthy participants expanded with aging, and the IVS, LVAW, and LVPW thickened, indicating the left ventricle remodeling with the progress of aging (Figures 1H–K).

**TABLE 1:** Characteristics of 231 healthy participants.

Group	20–29 (*n* = 39)	30–39 (*n* = 56)	40–49 (*n* = 47)	50–59 (*n* = 42)	60∼ (*n* = 47)	*p*
Gender, male (%)	25 (64.10)	29 (51.79)	30 (63.83)	23 (54.76)	28 (59.57)	NS
Height mean ± SD (cm)	171.03 ± 9.09	169.71 ± 9.15	169.34 ± 7.92	167.81 ± 8.49	165.26 ± 9.64	0.028
Weight mean ± SD (kg)	67.51 ± 11.74	69.91 ± 13.73	72.15 ± 11.70	73.55 ± 12.27	70.96 ± 10.75	NS
SBP mean ± SD (mmHg)	114.92 ± 15.37	116.54 ± 16.46	119.68 ± 14.33	117.50 ± 14.86	116.19 ± 14.53	NS
DBP mean ± SD (mmHg)	76.95 ± 13.12	80.63 ± 12.37	78.60 ± 13.50	80.50 ± 11.31	78.15 ± 11.70	NS
GLU mean ± SD (mmol/L)	5.06 ± 0.66	5.09 ± 0.64	5.02 ± 0.69	4.99 ± 0.69	4.94 ± 0.67	NS
CHO mean ± SD (mmol/L)	3.92 ± 0.72	4.15 ± 0.66	3.91 ± 0.68	3.86 ± 0.69	3.96 ± 0.65	NS
CRE mean ± SD (mmol/L)	76.49 ± 13.90	83.89 ± 15.77	81.89 ± 14.53	76.60 ± 15.84	81.66 ± 15.63	NS
BUN mean ± SD (mmol/L)	4.80 ± 1.08	5.06 ± 1.11	4.88 ± 1.06	5.06 ± 1.46	4.74 ± 1.48	NS
ALT mean ± SD (U/L)	30.44 ± 13.93	29.71 ± 15.14	29.36 ± 11.97	30.71 ± 11.95	30.09 ± 15.62	NS
AST mean ± SD (U/L)	30.15 ± 13.36	27.27 ± 10.60	29.28 ± 8.95	30.10 ± 8.05	29.96 ± 11.84	NS

### Serum H_2_S, Iron, and MDA Levels Change With Aging in Healthy Participants

**FIGURE 2:**
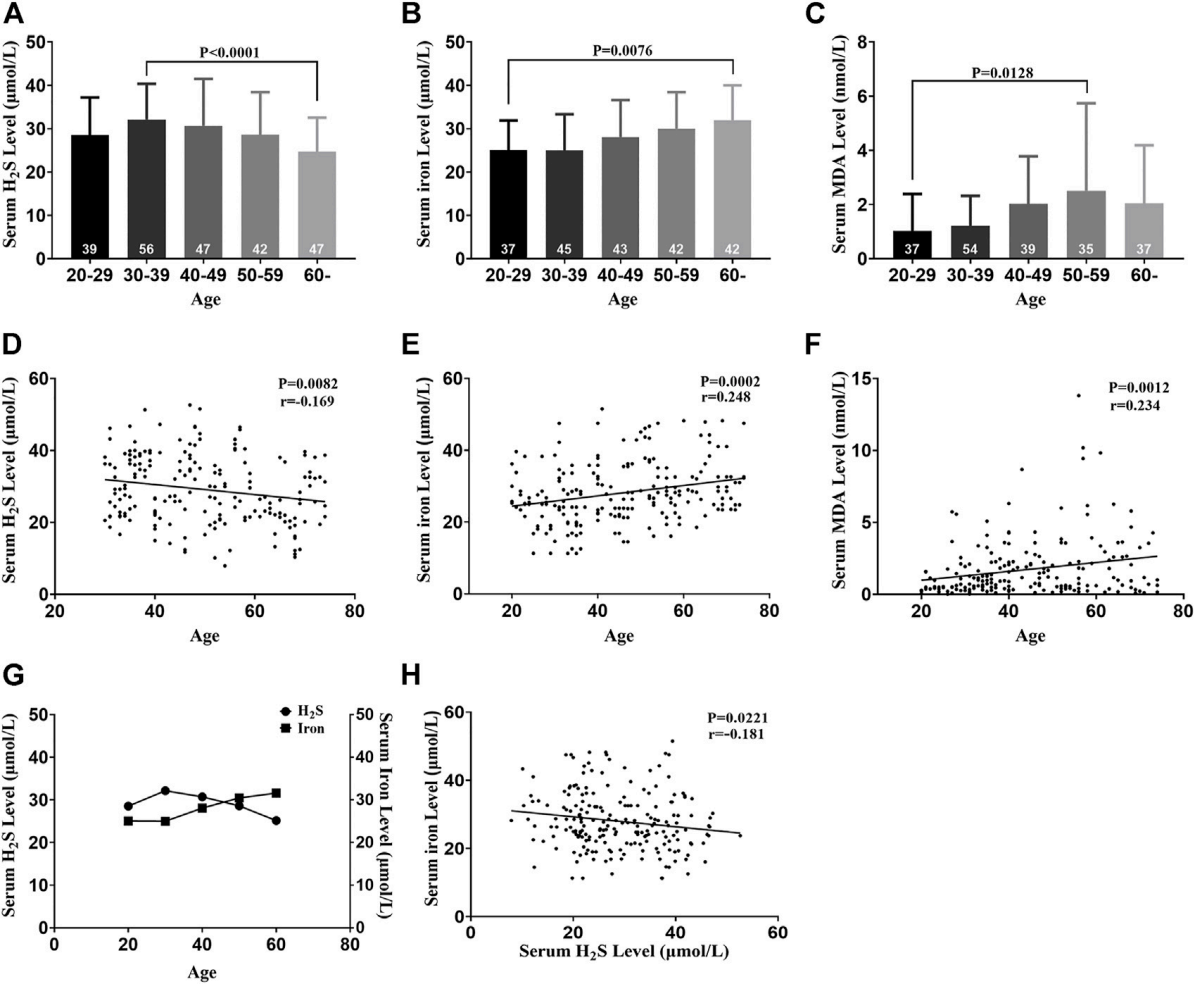
Serum H_2_S, iron, and MDA levels change with aging in healthy participants. **(A)** Serum H_2_S levels of five groups. **(B)** Serum iron levels of five groups. **(C)** Serum MDA levels of five groups. **(D)** Serum H_2_S level was negatively correlated with age. **(E)** Serum iron level was positively correlated with age. **(F)** Serum MDA level was positively correlated with age. **(G)** Serum iron and H_2_S levels changed with aging. **(H)** Serum iron level was negatively correlated with the serum H_2_S level. Results are means ± SEM. A *p*-value of <0.05 was considered significant. MDA: malondialdehyde.

Serum H_2_S levels in healthy participants decreased with aging and peaked in the 30–39 age groups, indicating that serum H_2_S negatively correlated with age (Figures 2A,D). Serum iron levels increased with aging, indicating that serum iron positively correlated with age (Figures 2B,E). Serum MDA increased with aging, indicating that serum MDA positively correlated with age (Figures 2C,F). Serum iron levels increased when serum H_2_S levels decreased, indicating a negative correlation between serum iron and the H_2_S level (Figures 2G,H).

### Exogenous H_2_S Enhanced H_2_S Synthase Expression in the Myocardial Tissue of Aging Rats

**FIGURE 3:**
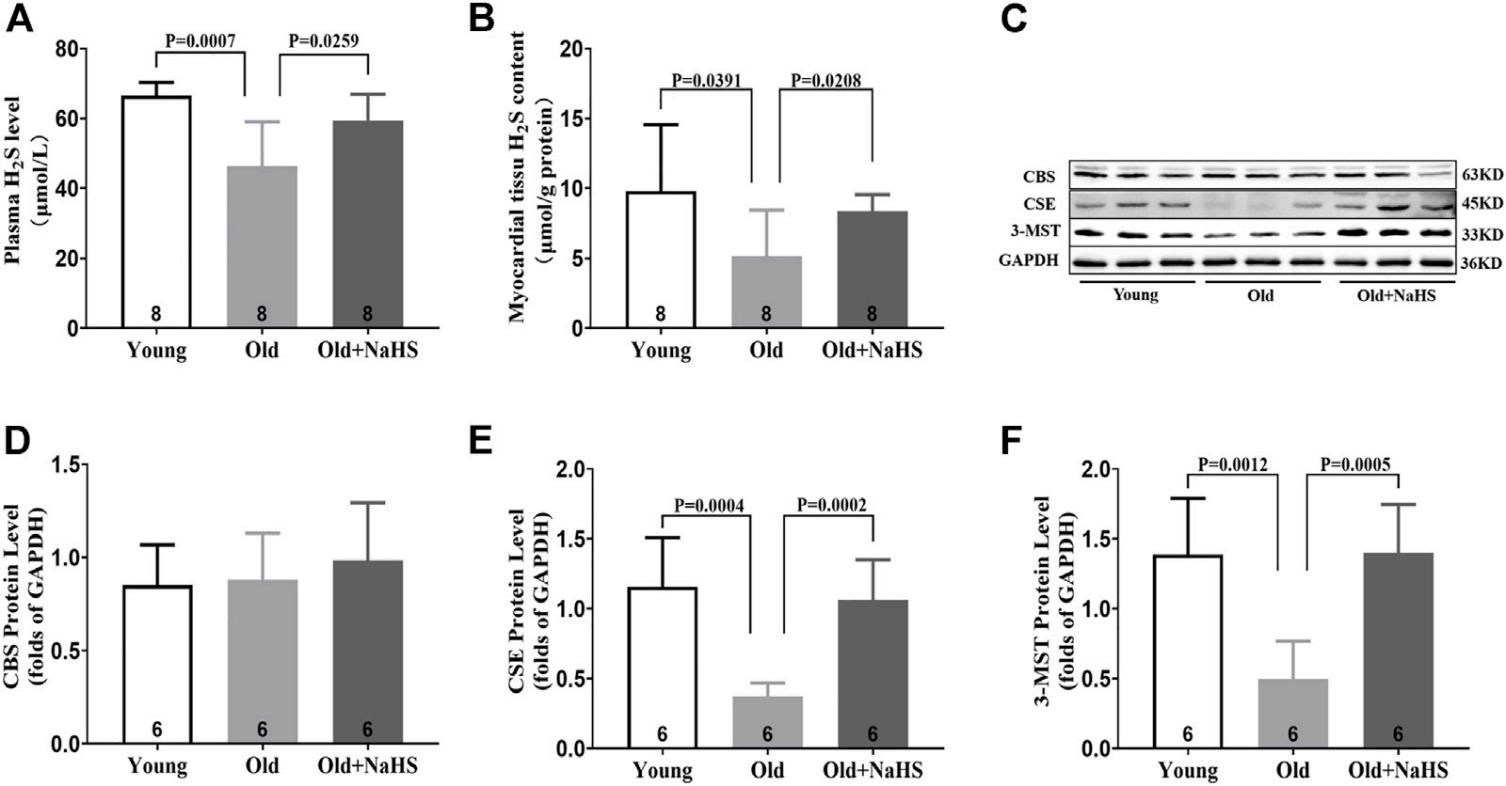
Exogenous H_2_S enhanced H_2_S synthase expression in the myocardial tissue of aging rats. **(A)** H_2_S levels in the plasma of aging rats. **(B)** H_2_S levels in the myocardial tissue of aging rats. **(C)** Representative Western blots for CBS, CSE, and 3-MST expressions in myocardial tissues of aging rats. GAPDH was used as the internal control. **(D)** Quantitative analysis for CBS expression in the myocardial tissue of aging rats. **(E)** Quantitative analysis for the CSE expression in the myocardial tissue of aging rats. **(F)** Quantitative analysis for the 3-MST expression in the myocardial tissue of aging rats. Results are means ± SEM. A *p*-value of <0.05 was considered significant.

H_2_S in the plasma and myocardial tissue of aging rats decreased compared with that of young rats. Treatment with NaHS increased the endogenous H_2_S levels (Figures 3A,B). The expression of endogenous H_2_S synthase CSE and 3-MST decreased in the myocardial tissue of aging rats. Treatment with NaHS enhanced the expression of endogenous H_2_S synthase CSE and 3-MST, but no significant differences in CBS expression were detected (Figures 3C–F).

### H_2_S Improved Cardiac Function in Aging Rats

**FIGURE 4:**
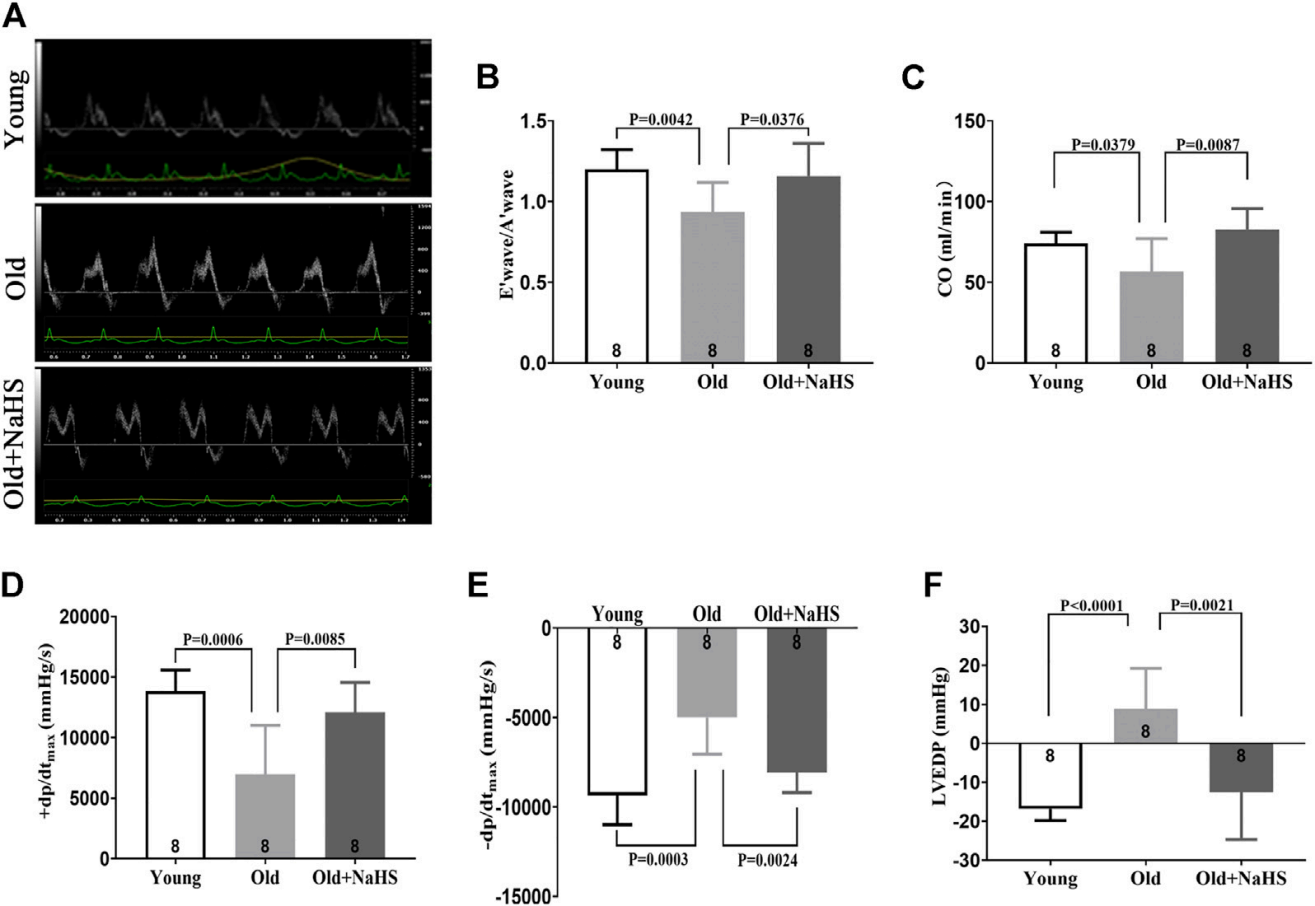
H_2_S improved cardiac function in aging rats. **(A)** Representative images of the PW Doppler-mode by echocardiography indicated E′ wave and A′ wave in aging rats. **(B)** E′ wave/A′ wave ratios in aging rats. **(C)** Values of CO in aging rats. **(D)** Values of +dp/dt_max_ in aging rats. **(E)** Values of −dp/dt_max_ in aging rats. **(F)** Values of LVEDP in aging rats. Results are means ± SEM. A *p*-value of <0.05 was considered significant. +dp/dtmax: the maximum rate of left ventricular pressure rise. −dp/dtmax: the maximum rate of left ventricular pressure fall. LVEDP: left ventricular end diastolic pressure.

According to the echocardiography results, the E′ wave was higher than A′ wave in young rats, and the E′ wave was lower than A′ wave in aging rats (Figure 4A). The E′ wave/A′ wave ratio was lower in aging rats (Figure 4B). Treatment with NaHS increased the E′ wave/A′ wave ratio in aging rats (Figures 4A,B). CO decreased in aging rats compared with that in young rats, and treatment with NaHS increased CO in aging rats (Figure 4C). The hemodynamics evaluation of +dp/dt_max_ and −dp/dt_max_ was lower, and LVEDP was higher in aging rats, indicating that the cardiac function significantly decreased in aging rats compared with that in young rats. Treatment with NaHS increased + dp/dt_max_ and −dp/dt_max_ and lowered LVEDP in aging rats (Figures 4D–F).

### H_2_S Alleviated Myocardial Injury in Aging Rats

**FIGURE 5:**
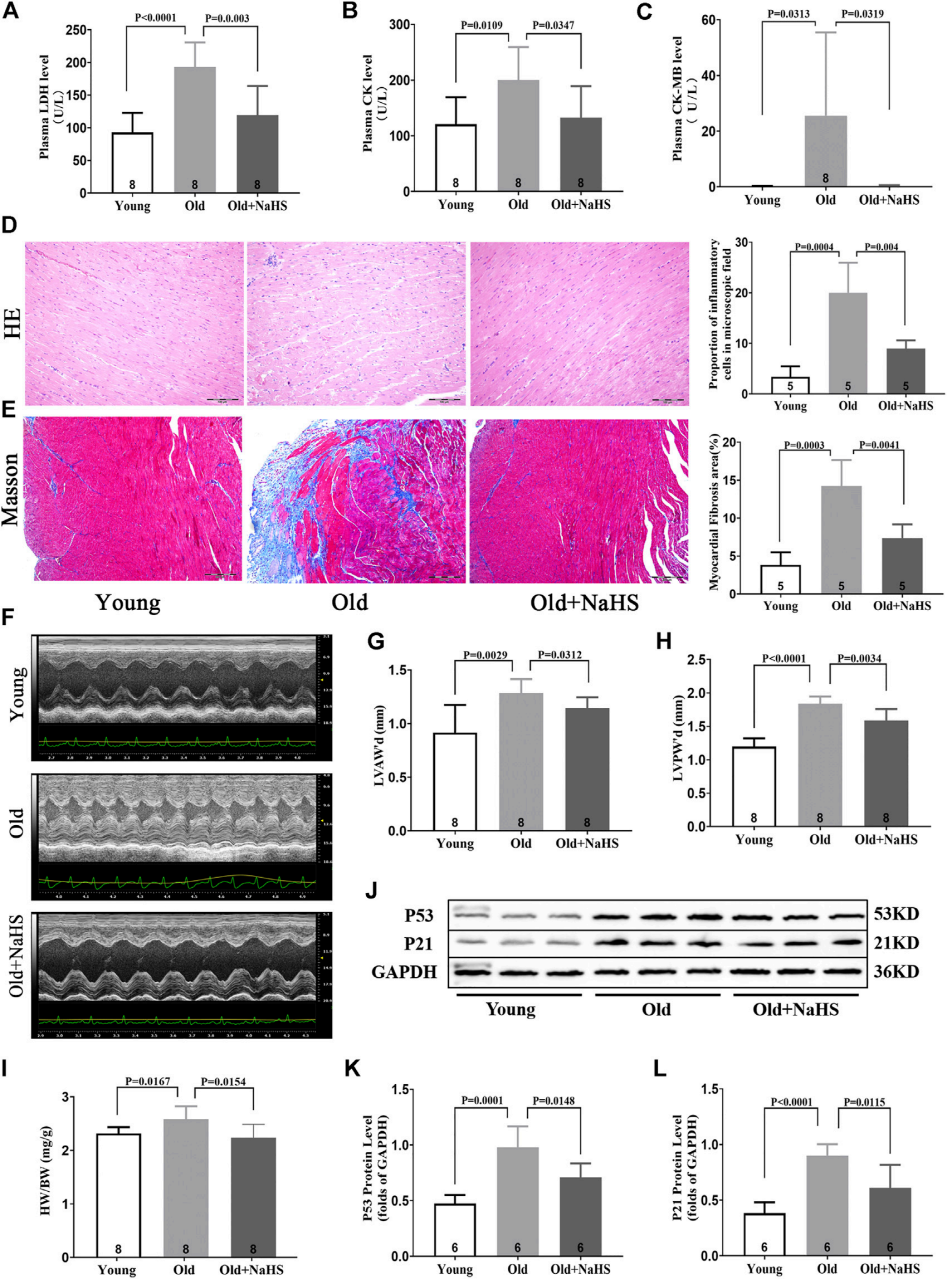
H_2_S alleviated myocardial injury in aging rats. **(A)** Levels of plasma LDH in aging rats. **(B)** Levels of plasma CK in aging rats. **(C)** Levels of plasma CK-MB in aging rats. **(D)** Representative HE-stained myocardial sections in aging rats and quantitative data on inflammatory or death cells in microscopic field (%). **(E)** Representative Masson-stained myocardial sections in aging rats and quantitative data on myocardial fibrosis (%). **(F)** Representative images of M-mode by echocardiography indicated LVAW and LVPW in aging rats. **(G)** Values of LVAW’d in aging rats. **(H)** Values of LVPW’d in aging rats. **(I)** HW/BW ratios in aging rats. **(J)** Representative Western blots for P53 and P21 expressions in myocardial tissues of aging rats. GAPDH was used as the internal control. **(K)** Quantitative analysis for the P53 expression in the myocardial tissue of aging rats. **(L)** Quantitative analysis for the P21 expression in the myocardial tissue of aging rats. Results are means ± SEM. A *p*-value of <0.05 was considered significant. LDH: lactate dehydrogenase. CK: creatine kinase. CK-MB: creatine kinase isoenzyme-MB. HW/BW: heart weight/body weight ratios.

Serum myocardial injury markers, including elevated LDH, CK, and CK-MB levels decreased significantly in response to NaHS treatment in aging rats (Figures 5A–C). The HE staining showed that the myocardial tissue structure in young rats was dense, and the myocardial fibers were intact. The number of inflammatory cells in the myocardial interstitium was significantly lower in young rats than the number in aging rats. The myocardial tissue in aging rats exhibited severe cell alignment disorders and more cardiomyocyte inflammatory infiltration and death. Treatment with NaHS significantly decreased the number of inflammatory cells in the myocardial interstitium and inhibited cardiomyocyte death in aging rats compared with the myocardium of untreated aging rats (Figure 5D).

The Masson staining showed that myocardial fibrosis increased in aging rats compared with that in young rats, and treatment with NaHS reduced myocardial fibrosis in aging rats (Figure 5E). The HW/BW ratio of aging rats increased, and M-mode images by echocardiography showed that the values of LVAW’d and LVPW’d increased compared with these parameters in young rats. Treatment with NaHS lowered the HW/BW ratio and inhibited LV remodeling in aging rats compared with that in untreated aging rats (Figures 5F–I).

Western blot analysis showed that treatment with NaHS reduced the protein expressions of p53 and p21 in myocardial tissue of aging rats and reduced the cardiac senescence phenomenon of aging rats (Figures 5J–L).

### H_2_S Inhibited Cardiomyocyte Ferroptosis in Aging Rats

**FIGURE 6:**
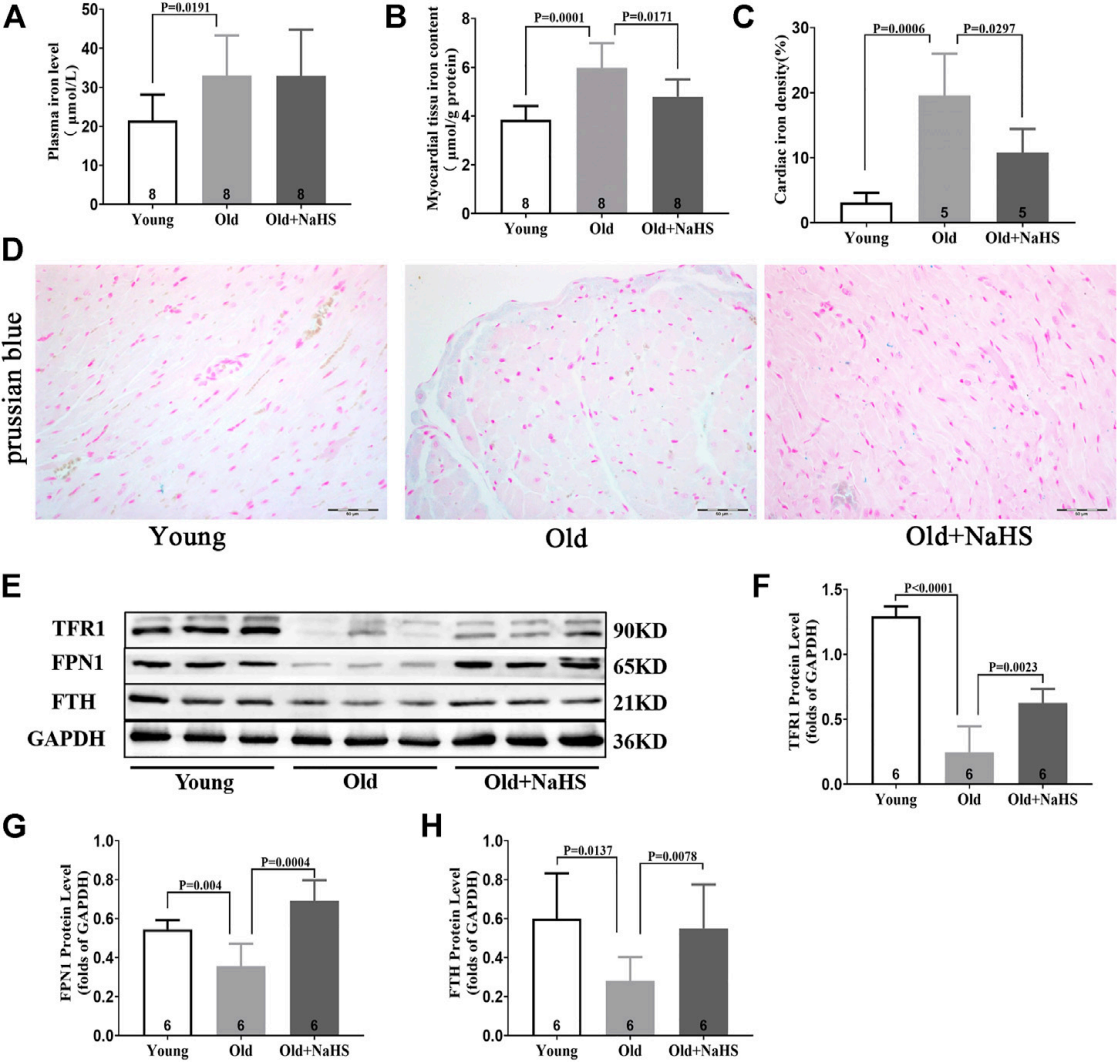
H_2_S regulated iron metabolism and reduced iron accumulation in aging rats. **(A)** Iron levels in the plasma of aging rats. **(B)** Iron levels in the myocardial tissue of aging rats. **(C)** Quantitative data on the Prussian-blue-iron-stained in aging rats. **(D)** Representative Prussian-blue-iron-stained in myocardial tissue sections of aging rats. **(E)** Representative Western blots for TFR1, FPN1, and FTH expressions in myocardial tissues. GAPDH was used as the internal control. **(F)** Quantitative analysis for the TFR1 expression in the myocardial tissue of aging rats. **(G)** Quantitative analysis for the FPN1 expression in the myocardial tissue of aging rats. **(H)** Quantitative analysis for the FTH expression in the myocardial tissue of aging rats. Results are means ± SEM. A *p*-value of <0.05 was considered significant.

Iron levels in the plasma and myocardial tissue of aging rats increased compared with those of young rats. The proportion of Prussian blue iron-stained cells in the myocardial tissue increased in aging rats. Treatment with NaHS reduced iron levels in the plasma and myocardial tissue and the proportion of Prussian blue iron-stained cells in the myocardial tissue of aging rats (Figures 6A–D). Western blot analyses showed that compared with young rats, the expressions of TFR1, FPN1, and FTH in the myocardial tissue of aging rats were attenuated compared with those of young rats. Treatment with NaHS partially enhanced the expression of iron metabolism regulatory proteins (Figures 6E–H).

**FIGURE 7:**
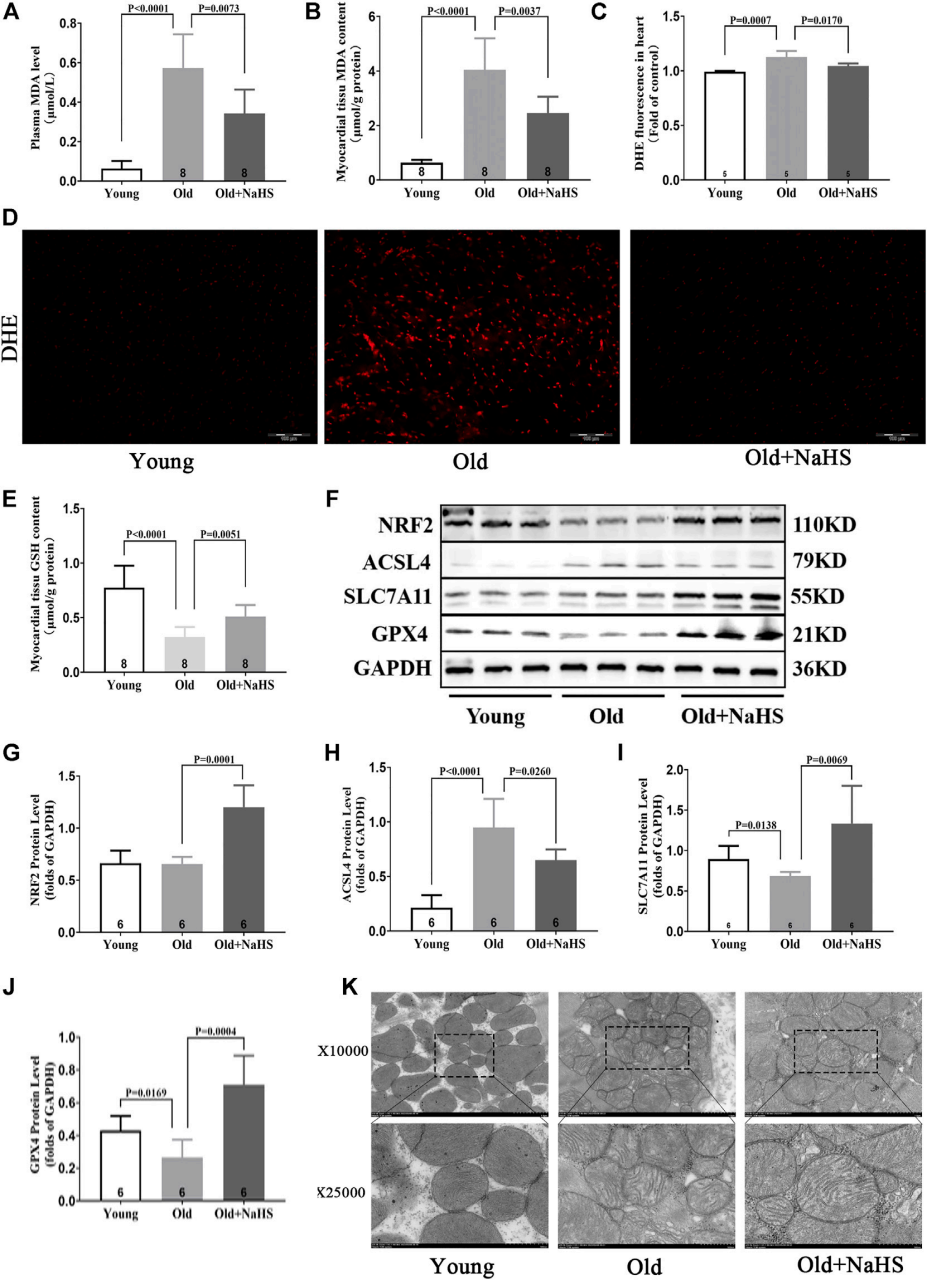
H_2_S inhibited cardiomyocyte ferroptosis in aging rats. **(A)** MDA levels in the plasma of aging rats. **(B)** MDA levels in the myocardial tissue of aging rats. **(C)** Quantitative data on DHE-fluorescence in aging rats. **(D)** Representative DHE-fluorescence in the myocardial tissue sections of aging rats. **(E)** GSH levels in the myocardial tissue of aging rats. **(F)** Representative Western blots for NRF2, ACSL4, SLC7A11, and GPX4 expressions in the myocardial tissues of aging rats. GAPDH was used as the internal control. **(G)** The quantitative analysis for NRF2 expression in the myocardial tissue of aging rats. **(H)** Quantitative analysis for ACSL4 expression in the myocardial tissue of aging rats. **(I)** Quantitative analysis for the SLC7A11 expression in the myocardial tissue of aging rats. **(J)** Quantitative analysis for the GPX4 expression in the myocardial tissue of aging rats. **(K)** Representative images of transmission electron microscopy for mitochondria in the cardiomyocyte of aging rats. Results are means ± SEM. A *p*-value of <0.05 was considered significant.

Compared with young rats, the levels of MDA in plasma and myocardial tissue of aging rats increased, and the fluorescence intensity of DHE in the myocardial tissue increased. NaHS treatment lowered the levels of MDA in plasma and myocardial tissue and reduced the fluorescence intensity of DHE (Figures 7A–D). The GSH level in the myocardial tissue of aging rats was lower than the GSH levels in young rats, and treatment with NaHS promoted the generation of GSH (Figure 7E); Western blot analyses showed that SLC7A11 and GPX4 expressions decreased, and the ACSL4 expression was enhanced in the myocardial tissue of aging rats compared with the expression in young rats. Treatment with NaHS enhanced NRF2 SLC7A11 and GPX4 protein expressions and attenuated the expression of ACSL4 (Figures 7F–J). Electron microscopy of cardiomyocytes showed that compared with young rats, the density of mitochondrial membranes increased, the mitochondrial cristae decreased or disappeared, the outer membranes of mitochondria were broken, and mitochondria edema was observed in aging rats compared with that in young rats. Treatment with NaHS inhibited the abnormal changes in cardiomyocyte mitochondria (Figure 7K). Based on these findings, we cautiously surmised that H_2_S inhibited cardiomyocyte ferroptosis.

### H_2_S Alleviated Myocardial Injury and Improved Cardiac Function in Rats Fed With a HID

To verify whether H_2_S could regulate iron metabolism, we used a HID to induce iron-overload cardiomyopathy in rats. Aging rats began to die on the eighth of HID feeding. By the 10th week, all 12 aging rats on the HID died. Meanwhile, seven aging rats in the old + HID + NaHS group died and five survived, with a mortality rate of 58.3%. Three aging rats in the old + NID group died, and nine survived, with a mortality rate of 25%. These findings indicated that HID accelerated the death of aging rats (Supplementary Figures S2A,B).

HID did not affect H_2_S levels in the plasma and myocardial tissue of young rats, and no significant differences in the expression of endogenous H_2_S synthase CSE, CBS, and 3-MST were detected in the myocardial tissue. However, treatment with NaHS enhanced the expression of endogenous H_2_S synthase CSE, CBS, and 3-MST in the myocardial tissue of young rats fed with HID and increased H_2_S levels in plasma and myocardial tissue compared with the H_2_S levels in untreated rats fed with a HID (Supplementary Figures S3A–F).

**FIGURE 8:**
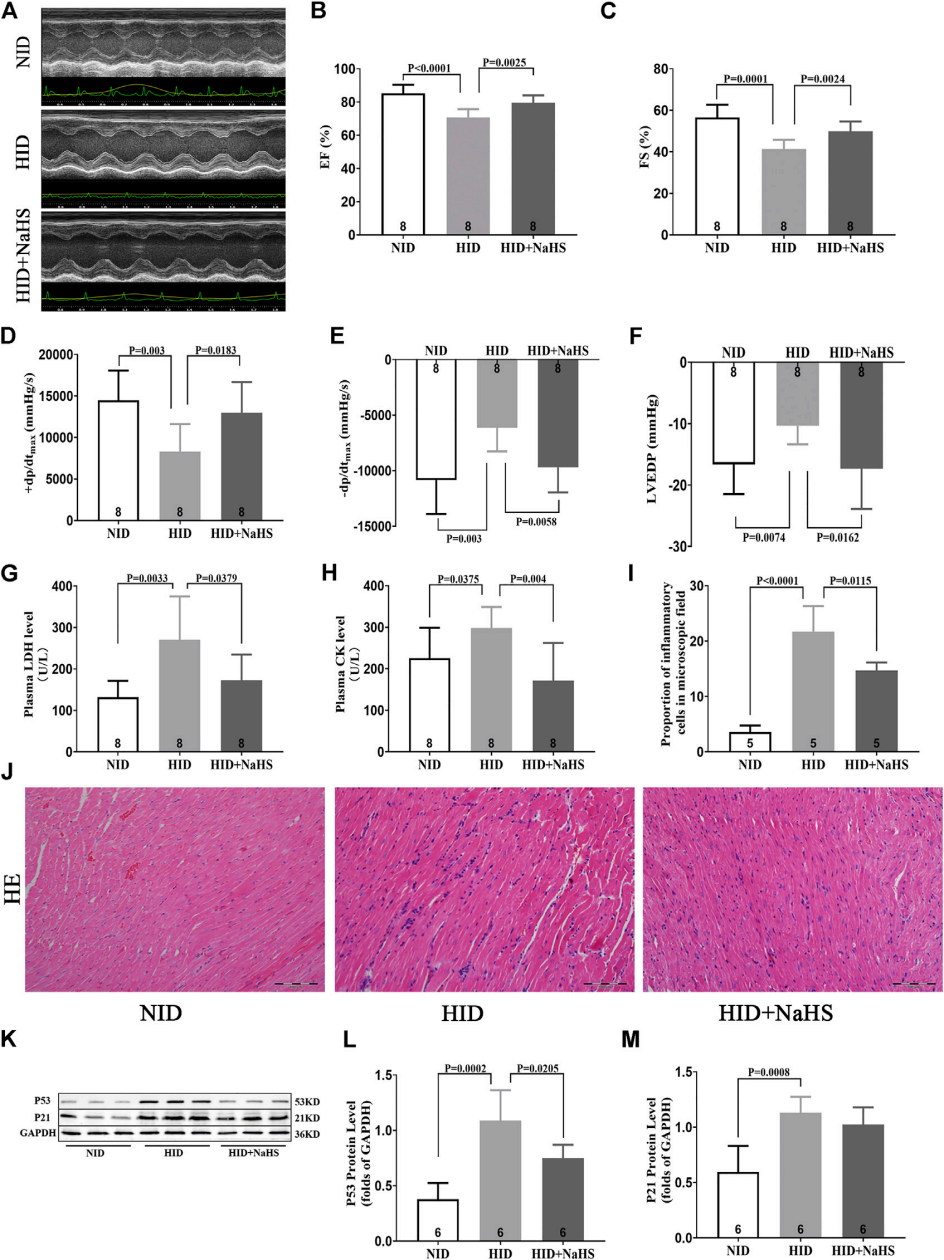
H_2_S alleviated myocardial injury and improved cardiac function in rats fed with HID. **(A)** Representative images of M-mode by echocardiography in HID rats. **(B)** Values of EF (%) in HID rats. **(C)** Values of FS (%) in HID rats. **(D)** Values of +dp/dt_max_ in HID rats. **(E)** Values of −dp/dt_max_ in HID rats. **(F)** Values of LVEDP in HID rats. **(G)** Levels of plasma LDH in HID rats. **(H)** Levels of plasma CK in HID rats. **(I)** Quantitative data on inflammatory cells in the microscopic field (%) of HID rats. **(J)** Representative HE-stained myocardial sections of HID rats. **(K)** Representative Western blots for P53 and P21 expressions in myocardial tissues of HID rats. GAPDH was used as the internal control. **(L)** Quantitative analysis for the P53 expression in the myocardial tissue of HID rats. **(M)** Quantitative analysis for the P21 expression in the myocardial tissue of HID rats. Results are means ± SEM. A *p*-value of <0.05 was considered significant.

Feeding HID reduced the cardiac function in young rats, which was mainly manifested by the decrease in EF%, FS%, +dp/dt_max_, and −dp/dt_max_ and increased LVEDP. Treatment with NaHS restored EF%, FS%, +dp/dt_max_, and −dp/dt_max_ and reduced LVEDP (Figures 8A–F).

Serum LDH and CK increased in rats fed with HID, and treatment with NaHS significantly reduced the levels of serum myocardial injury biomarkers (Figures 8G,H). HE staining showed that cardiomyocytes in rats fed with NID were dense, and the number of inflammatory cells in the myocardial interstitium was significantly lower than rats fed with HID. Treatment with NaHS decreased the number of inflammatory cells in the myocardial interstitium (Figures 8I,J). However, no significant differences in the area of myocardial fibrosis were detected between the groups (Supplementary Figure S4). Western blot analysis showed that HID enhanced the expression of senescence marker proteins p53 and P21, indicating that the cardiac senescence increased and accelerated the aging process in rats. Treatment with NaHS reduced the protein expression of p53 in the myocardial tissue of rats fed with a HID, indicating that cardiac senescence was reduced (Figures 8K–M).

**FIGURE 9:**
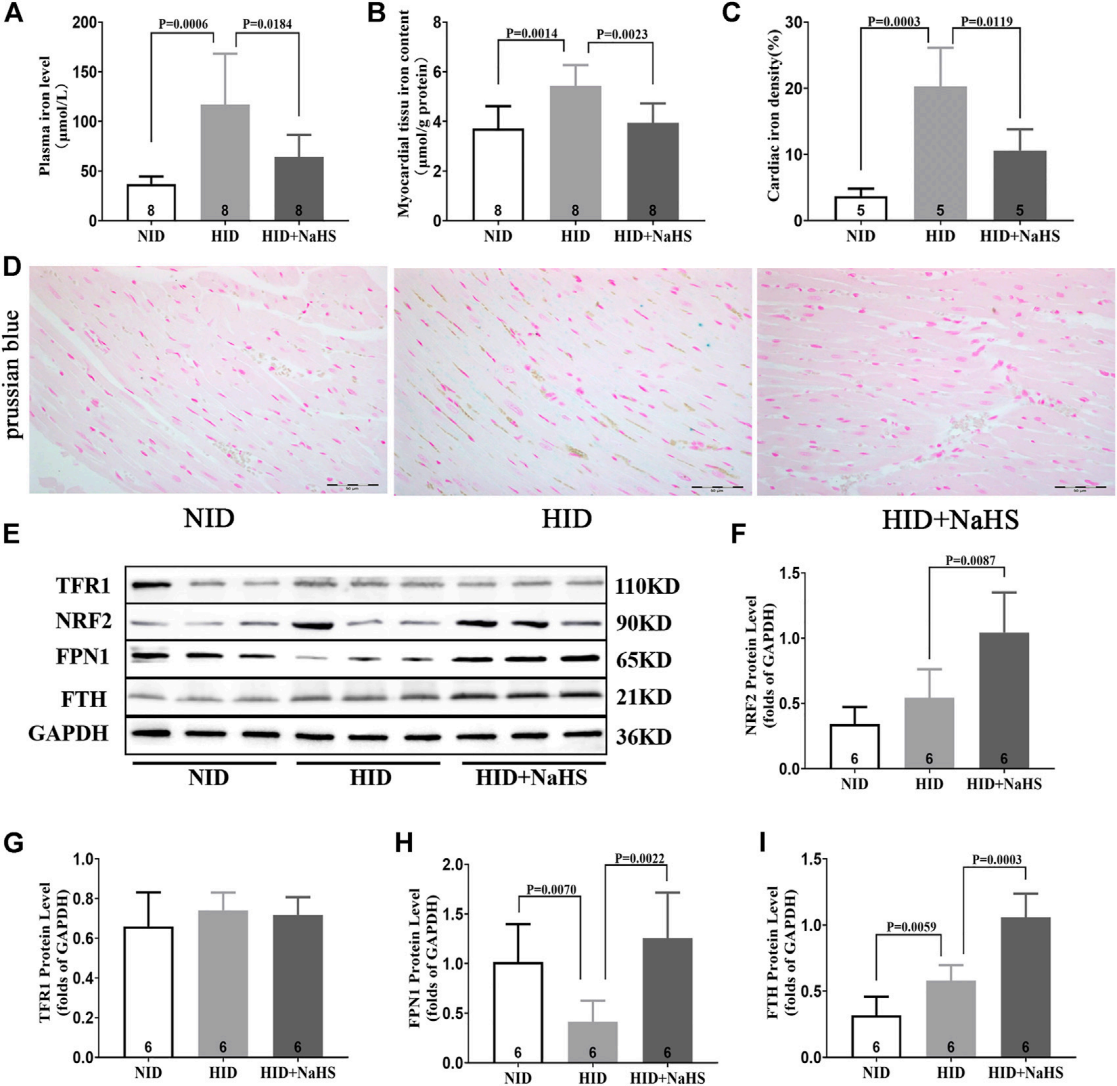
H_2_S regulated cardiomyocyte iron metabolism in rats fed with HID. **(A)** Iron levels in the plasma of HID rats. **(B)** Iron levels in the myocardial tissue of HID rats. **(C)** Quantitative data on Prussian-blue staining in HID rats. **(D)** Representative Prussian-blue staining in myocardial tissue sections of HID rats. **(E)** Representative Western blots for TFR1, NRF2, FPN1, and FTH expressions in the myocardial tissues of HID rats. GAPDH was used as the internal control. **(F)** Quantitative analysis for NRF2 expression in the myocardial tissue of HID rats. **(G)** Quantitative analysis for the TFR1 expression in the myocardial tissue of HID rats. **(H)** Quantitative analysis for the FPN1 expression in the myocardial tissue of HID rats. **(I)** Quantitative analysis for the FTH expression in the myocardial tissue of HID rats. Results are means ± SEM. A *p*-value of <0.05 was considered significant.

Iron levels in the plasma and myocardial tissue and the proportion of iron-stained cells in the myocardial tissue of rats fed with a HID increased. Treatment with NaHS lowered the levels of iron in plasma and myocardial tissue and reduced the proportion of iron-stained cells in the myocardial tissue (Figures 9A–D). Western blot analysis showed that NaHS enhanced the expressions of NRF2, FPN1, and FTH in the myocardial tissue of rats fed with HID, but no significant differences in the expression of TFR1 were detected (Figures 9E–I).

## Discussion

Age is a major risk factor for cardiac dysfunction and overall cardiovascular disease. Greater time for exposure to injurious stimuli, such as hypertension, metabolic stress, or ischemic injury, clearly contributes to cardiac dysfunction with aging. The aging heart has limited endogenous capacity for repair or regeneration. Thus, aging impairs cardiac reserves and elevates the risk of cardiac dysfunction.

In our clinical experiments, echocardiographic measurements indicated that structural and functional changes contributing to cardiac dysfunction occurred in the aging heart, even in the absence of overt injury or disease. Normal aging is generally accompanied by thickening and stiffening of the LV walls and the interventricular septum. In addition, the LV diameter and cardiac fibrosis increase. These findings are consistent with previous studies (Fleg et al., 2015). However, our study also demonstrates that the abnormal cardiac diastolic function usually occurred before changes in the cardiac systolic function during aging. This may be due to the slow degeneration and death of cardiomyocytes in the aging process. Compensatory mechanisms cause fibrosis-like changes in the ventricular wall, specifically manifested as increased ventricular stiffness, limited ventricular diastolic capacity, insufficient filling times, and cardiac dysfunction. Thus, although EF%, FS%, and SV did not change, E/A, E/e', E', and LAVI, which measured cardiac diastolic function, were abnormal. This “diastolic heart failure” with preserved cardiac systolic function (Francesco et al., 2013) was verified in aging rats (Figures 4A,B). At the same time, centripetal hypertrophy of the left ventricle occurred, resulting in LV lumen stenosis (Figures 5F–I). Limited filling of the heart during diastole leads to less effective circulating blood, cardiac dysfunction, and even fatal heart failure.

Iron is an essential element and is fundamental to many biological processes, including oxygen transport and storage and oxidative phosphorylation and redox reactions due to its ability to transport oxygen and electrons (Coffey and Ganz, 2017). Therefore, iron metabolism disorders, especially iron excess, result in abnormal oxidation–reduction reactions, which contribute to organ dysfunction *via* the accumulation of ROS (Gudjoncik et al., 2014). Our results showed that serum iron and MDA increased with aging and with decreased serum H_2_S levels. Thus, serum iron and MDA negatively correlated with the level of serum H_2_S in healthy individuals. The decrease in endogenous H_2_S levels aggravates the progress of cardiovascular diseases (Testai et al., 2021). However, the relationship between endogenous H_2_S, aging, and cardiac dysfunction in healthy individuals has not been reported yet. For the first time, we found that endogenous H_2_S levels decreased with aging in healthy individuals, and the decreased H_2_S was accompanied by cardiac dysfunction.

The animal experiments were consistent with the clinical experiments. In aging rats, endogenous H_2_S levels decreased, oxidative stress increased, iron accumulated, myocardial injury and myocardial fibrous tissue hyperplasia increased, and cardiac function declined. After treatment with exogenous H_2_S, H_2_S in plasma and myocardial tissue increased, the expression of endogenous H_2_S synthase was enhanced in the myocardial tissue, the levels of oxidative stress and iron in plasma and myocardial tissue decreased, myocardial injury was attenuated, and cardiac function improved. The mechanism for the enhanced endogenous H_2_S by exogenous H_2_S supplementation seems to be the enhanced CSE expression at a certain range of H_2_S concentration, and the appropriate concentration of exogenous H_2_S affects CSE transcription and translation (Yan, 2004; Wang, 2013). The increased expression of mitochondrial 3-MST in response to exogenous H_2_S supplementation may be *via* the reduction in mitochondrial damage in cardiomyocytes (Li et al., 2016b). The heart contains several kinds of cells, and H_2_S can be synthesized by cells other than cardiomyocytes; thus, CBS is also expressed in the myocardial tissue. The appropriate concentration of exogenous H_2_S supplementation can promote the expression of CBS in myocardial tissue (Shyam Sundar Nandi, 2017). However, the expression of endogenous H_2_S synthase in the two animal models constructed in our study was slightly different, which conflicted with previous conclusions. These differences may be due to the different animal species and animal model conditions.

Cardiac dysfunction was caused by iron accumulation in aging and HID animal models, consistent with several previous studies. However, the previous studies focused on the increased oxidative stress induced by iron accumulation and the effects of iron accumulation on cardiac electrophysiological conduction (Wongjaikam et al., 2016; Wongjaikam and KumfuS, 2017). Our study demonstrated that iron accumulation increased oxidative stress levels and caused cardiac dysfunction, which may be partly caused by cardiomyocyte ferroptosis and aggravated by cardiac senescence. Iron metabolism in cardiomyocytes is mainly regulated by FPN1, TFR1, and FTH (Muckenthaler et al., 2017). We found that the expressions of TFR1, FTH, and FPN1 were lower in myocardial cells of aging rats than the expression in young rats. These effects in aging rats may be caused by the decreased ability to regulate iron metabolism and maintain iron homeostasis. In aged cardiomyocytes, increases in autophagic flow and autocrine effects of hepcidin promote the degradation of FPN1 and FTH, therefore reducing the expression of these two proteins (Gao et al., 2016; Hou et al., 2016; Zhang et al., 2019). In most types of cells, the control of TFR1 expression is mediated by IRP1 *via* interactions with iron-responsive elements (Muckenthaler et al., 2017). The reduced expression of FPN1 leads to decreased iron release out of the cells, and autophagy of FTH increases intracellular iron, leading to the accumulation of iron inside of the cells. These can downregulate IRP1 expression, resulting in decreased TFR1 expression.

Exogenous H_2_S supplementation affected the expression of several key iron metabolism regulatory proteins and reduced cardiac senescence similar to previous studies (Wang et al., 2021). However, our study also demonstrated the effects of H_2_S on the expressions of FTH and FPN1 in cardiomyocytes. FPN1 is the only known mammalian iron export protein, and FTH is the main iron storage protein in cells (Tian et al., 2021; Wang et al., 2022). Increasing FPN1 expression and inhibiting the degradation of FTH reduce intracellular iron accumulation (Gao et al., 2016; Hou et al., 2016). H_2_S inhibits ferritinophagy signaling by activating NRF2 and PPAR-γ, which suppress ferroptosis (Wang et al., 2021; Wang et al., 2022). In our study, the NRF2 protein expression also changed when the expressions of FTH and FPN1 were enhanced after exogenous H_2_S supplementation. The NRF2/FTH and NRF2/FPN1 axes have already confirmed (Liu et al., 2020; Han et al., 2021). Thus, we speculated that the factors led to altered FTH and FPN1 expressions. Changes in the TFR1 expression may be due to a decreased intracellular iron accumulation. Decreased FPN1 in HID-fed rats may be related to the increased autocrine function of hepcidin caused by the increased expression of p53 in myocardial tissue (Wang et al., 2019; Zhang et al., 2019), while the increased expression of FTH in HID-fed rats may be a positive feedback effect caused by cellular iron accumulation. However, further research is needed to explore the specific mechanisms and causal relationships.

Apart from iron-mediated oxidative stress, cells must also cope with a variety of ROS. The NRF2 pathway regulates not only several genes involved in iron metabolism but also the cellular antioxidant responses. H_2_S stabilizes NRF2 and induces NRF2 target genes through antioxidant/electrophilic response elements. The ability of H_2_S antioxidant stress to inhibit cell death is dependent on NRF2 (Hourihan, 2013). In aging rats, cardiac dysfunction was accompanied by decreased levels of H_2_S in plasma and myocardial tissue and elevated oxidative stress levels. In addition, the GSH level in myocardial tissue decreased, and GSH is synthesized by glutamate, glycine, and cysteine. Cysteine comes from the transformation of cystine, which is transported from the extracellular place into cells by system^XC−^ (Lin et al., 2015). The transmembrane transporter SLC7A11 is an important component of system^XC−^, and the expression of SLC7A11 is regulated by the upstream NRF2 pathway (Kimura et al., 2010). Pathways related to H_2_S antioxidant stress include the activation of the NRF2 pathway (Calvert et al., 2009). H_2_S also induces the reduction of cystine to cysteine, increases the intracellular content of cysteine as the substrate of GSH synthesis, and enhances the transport of cysteine (Kimura et al., 2010). Our findings indicated that exogenous H_2_S treatment in aging rats enhanced the expressions of NRF2, SLC7A11, and GPX4 and elevated GSH levels in myocardial tissue. GSH is a necessary substrate for GPX4 to exert antioxidant effects. These combined effects reduced oxidative stress levels, reduced myocardial injury, and improved cardiac function.

Ferroptosis is an iron-dependent cell death form and is triggered by the excessive accumulation of lipid peroxide (Dixon et al., 2012). The phenomena we observed in the aging rat model, including the accumulation of iron and the increase in the lipid metabolite MDA, were consistent with the characteristics of ferroptosis. ACSL4 plays an important role in the occurrence and development of ferroptosis by promoting the synthesis of lipid peroxide. Of note, ACSL4 can be used as an indicator of the ferroptosis program (Proneth et al., 2017; Stockwell et al., 2017). The expression of ACSL4 increased in both myocardial injury and aging organisms, which aggravated the accumulation of lipid peroxides (Proneth et al., 2017; Wang et al., 2021; Zhou et al., 2022). Exogenous H_2_S supplementation may inhibit the expression of ACSL4 by reducing myocardial injury and consequently inhibiting the synthesis of polyunsaturated fatty acids. GPX4 is a selenoprotein and is a unique intracellular antioxidant enzyme that can directly quench the metabolites of lipid peroxide in cell membranes (Shah et al., 2018; Li et al., 2020). Therefore, decreased GPX4 activity leads to ferroptosis. In our current research, the ACSL4 expression increased, and the expression of GPX4 decreased in the myocardial tissue of aging rats. The main subcellular organ attacked by ROS is mitochondria, and the abnormal changes in mitochondria showed by electron microscopy were consistent with the characteristics of ferroptosis. After treatment with exogenous H_2_S, the expression of GPX4 increased, the expression of ACSL4 decreased, and the abnormal changes in mitochondria were suppressed. Therefore, we speculate that the decline in endogenous H_2_S levels and the cardiomyocyte ferroptosis may be involved in the cardiac dysfunction of aging, and the decline in endogenous H_2_S levels aggravates cardiomyocyte ferroptosis.

We have only preliminarily confirmed that aging involves elevated oxidative stress levels, iron accumulation, and decreased endogenous H_2_S levels, accompanied by cardiac dysfunction and cardiac remodeling. The cardiac dysfunction and cardiac remodeling associated with aging are closely related to the decreased endogenous H_2_S levels, and ferroptosis participates in the pathological process of cardiac dysfunction and cardiac remodeling. However, H_2_S and ferroptosis agonists and inhibitors are not used *in vivo*. Therefore, the specific process of ferroptosis leading to cardiac dysfunction and the protective mechanism of H_2_S will be further studied.

We speculate that H_2_S may inhibit cardiomyocyte ferroptosis by reducing iron accumulation and the oxidative stress level to reduce the impact of aging on cardiac function. Targeted intervention in the endogenous H_2_S levels may protect cardiac function and reduce the risk of cardiovascular diseases in aging.

## Data Availability

The original contributions presented in the study are included in the article/Supplementary Material; further inquiries can be directed to the corresponding authors.
