# Supplementary material for: Hydrogen Sulfide Inhibits Ferroptosis in Cardiomyocytes to Protect Cardiac Function in Aging Rats
Source: Front Mol Biosci. 2022 Jul 22;9:947778. doi: 10.3389/fmolb.2022.947778 (PMC9355033; doi:10.3389/fmolb.2022.947778)
Supplement: Supplementary file 1 [file DataSheet1.PDF]

Supplementary Material

Supplementary Figures1

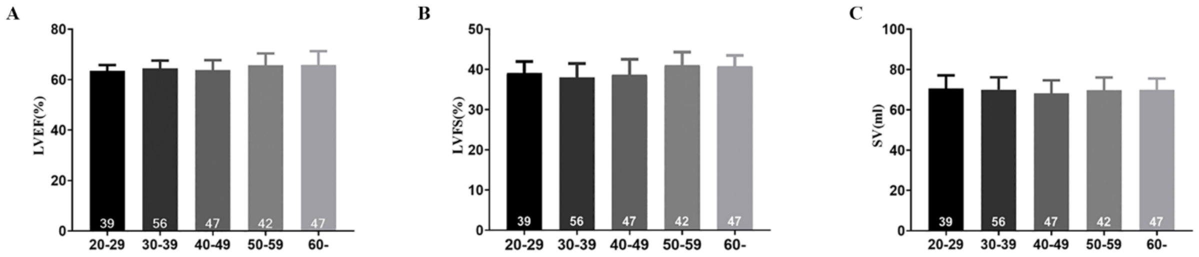

Supplementary Fig.1 With the progress of aging,the value of EF% and FS% did not decrease.

(A). The values of EF% in healthy of all age groups.**(B).**The values of FS% in healthy of all age groups.**(C).**The values of SV in healthy of all age groups.**EF%:**Left ventricular ejection fractio.**FS%:**Left ventricular short axis shortening rate.**SV:** Stroke output

SupplementaryFigures2

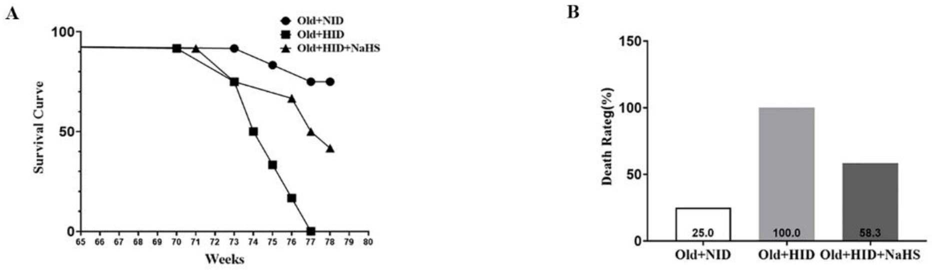

Supplementary Fig.2 HID can accelerate the death of aging rats

(A). Survival curve of aging rats in all group.**(B)**.Mortality of aging rats in all group

Supplementary Figures3

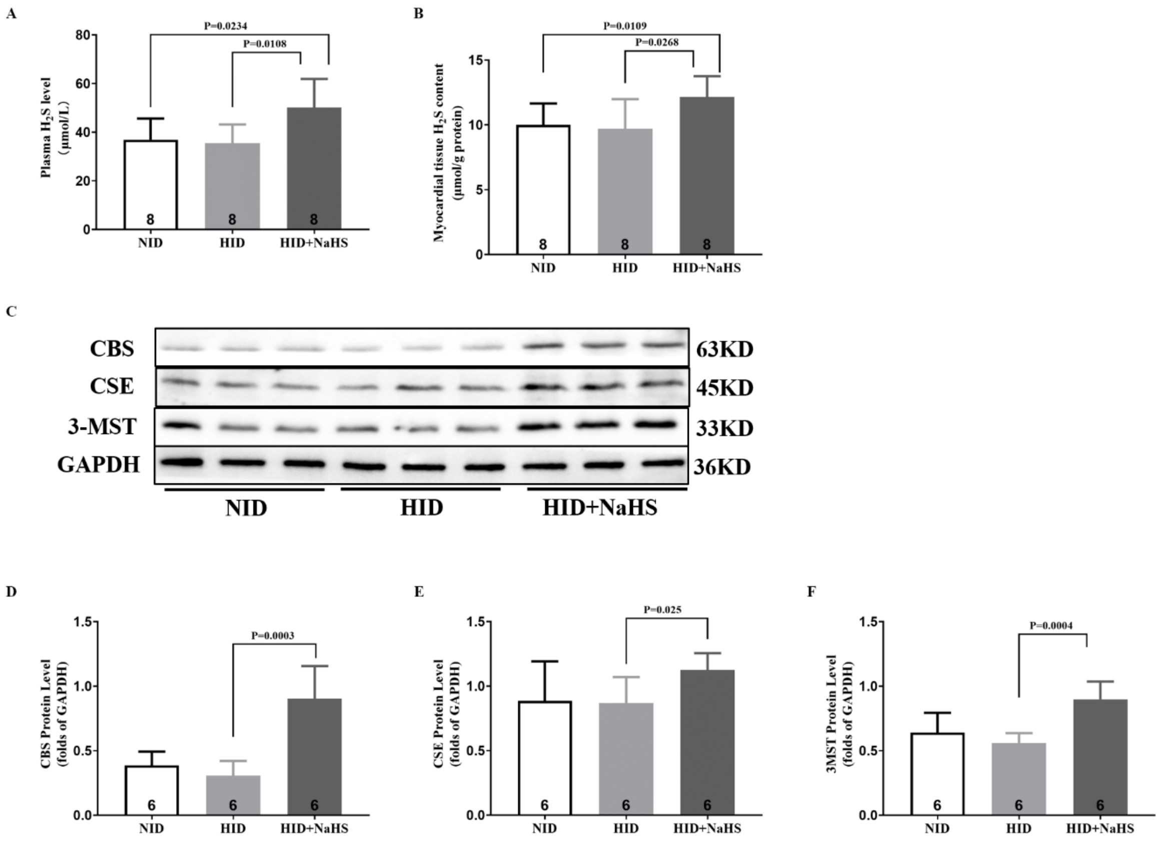

Supplementary Fig.3 Exogenous H_2S can enhance the expression of endogenous H_2S producing enzymes in myocardium of ratsfed with HID,and promote the production of endogenous H_2S.

(A-B). The H_2S levels in the plasma and myocardial tissue of all group rats.**(C).**Representative Western blots for CSE,3-MST and CBS expression in the myocardial tissues of all group rats.GAPDH was used as the internal control.**(D-F).**The quantitative analysis for CSE,3-MST and CBS expression in the myocardial tissue of all group rats.Results are means ± SEM. A P of <0.05 was considered significant

Supplementary Figures4

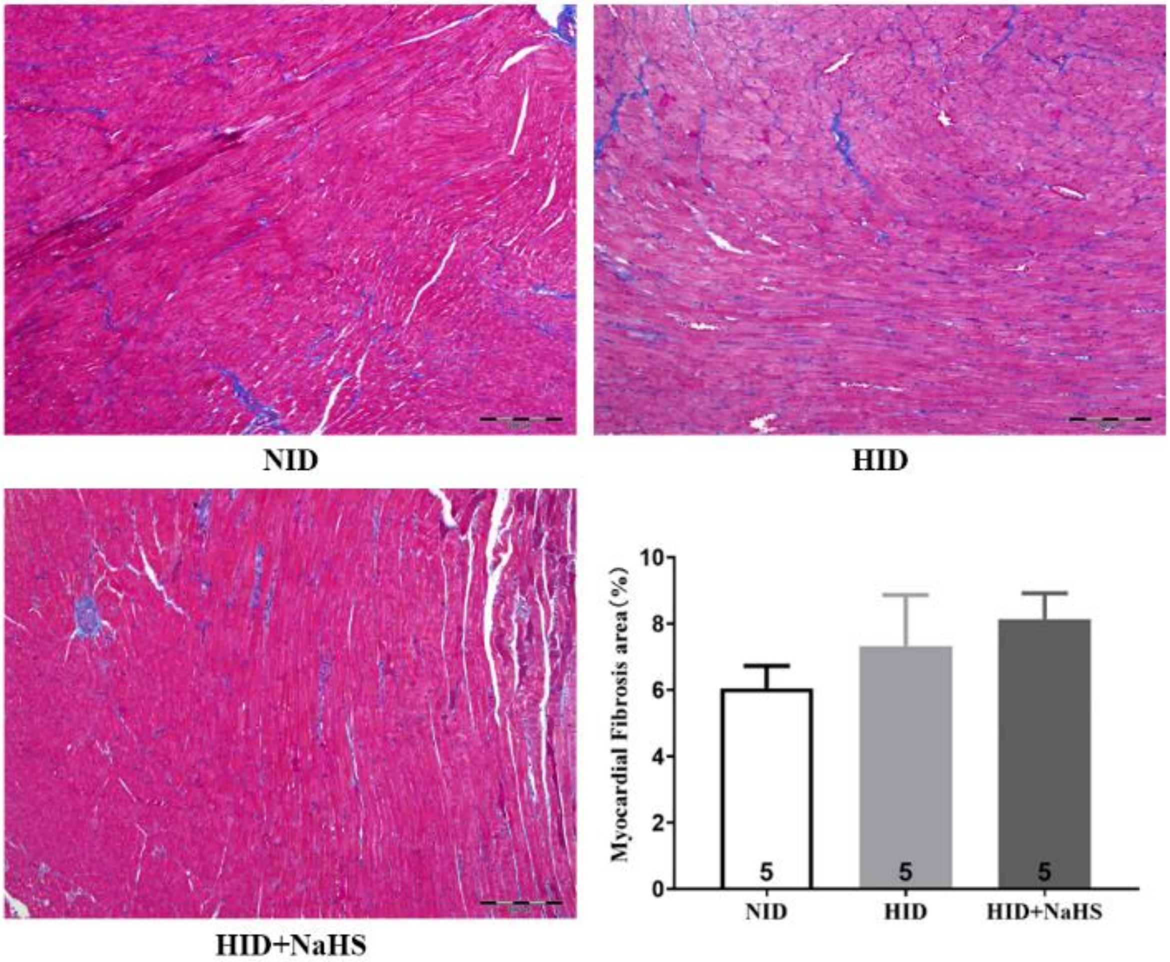

Supplementary Fig.4 Representative HE-stained myocardial sections of all group rats and quantitative data of inflammatory cells in microscopic field.
